# Impact of Rural Residence on Warfarin Use and Clinical Events in Patients with Non-Valvular Atrial Fibrillation: A Canadian Population Based Study

**DOI:** 10.1371/journal.pone.0140607

**Published:** 2015-10-14

**Authors:** Cynthia Wu, Michael Sean McMurtry, Roopinder K. Sandhu, Erik Youngson, Justin A. Ezekowitz, Padma Kaul, Finlay A. McAlister

**Affiliations:** 1 Department of Medicine, Division of Hematology, University of Alberta, Edmonton, Alberta, Canada; 2 Department of Medicine, Division of Cardiology, University of Alberta, Edmonton, Alberta, Canada; 3 Patient Health Outcomes Research and Clinical Effectiveness Unit, University of Alberta, Edmonton, Alberta, Canada; 4 Canadian VIGOUR Center, University of Alberta, Edmonton, Alberta, Canada; 5 Department of Medicine, Division of General Internal Medicine, University of Alberta, Edmonton, Alberta, Canada; Kurume University School of Medicine, JAPAN

## Abstract

**Background and Purpose:**

We studied whether anticoagulant use and outcomes differed between rural versus urban Canadian non-valvular atrial fibrillation (NVAF) patients prior to the introduction of direct oral anticoagulant drugs.

**Methods:**

Retrospective cohort study of 25,284 adult Albertans with NVAF between April 1, 1999 and December 31, 2008.

**Results:**

Compared to urban patients, rural patients were older (p = 0.0009) and had more comorbidities but lower bleeding risk at baseline. In the first year after NVAF diagnosis, urban patients were less likely to be hospitalized (aOR 0.82, 95%CI 0.77–0.89) or have an emergency department visit for any reason (aOR 0.61, 95%CI 0.56–0.66) but warfarin dispensation rates (72.2% vs 71.8% at 365 days, p = 0.98) and clinical outcomes were similar: 7.8% died in both groups, 3.2% rural vs. 2.8% urban had a stroke or systemic embolism (SSE) (aOR 0.92, 95%CI 0.77–1.11), and 6.6% vs. 5.7% (aOR 0.93, 95%CI 0.81–1.06) had a bleed. Baseline SSE risk did not impact warfarin dispensation (73.0% in those with high vs. 72.8% in those with low CHADS_2_ score, p = 0.85) but patients at higher baseline bleeding risk were less likely to be using warfarin (69.2% high vs. 73.6% low HASBLED score, p<0.0001) in the first 365 days after diagnosis. In warfarin users, bleeding was more frequent (7.5% vs 6.2%, aHR 1.51 [95%CI 1.33–1.72]) but death or SSE was less frequent (7.0% vs 18.1%, aHR 0.60 [0.54–0.66]).

**Conclusion:**

Warfarin use and clinical event rates did not differ between rural and urban NVAF patients in a universal access publically-funded healthcare system.

## Introduction

Non-valvular atrial fibrillation (NVAF) is associated with significant risk of stroke or systemic embolism (SSE) [1]. Temporal prescription trends, stroke/bleeding rates, and mortality statistics show a substantial proportion at high risk of SSE do not receive anticoagulants or are sub-optimally treated [2–4].

The impact of rural residence on anticoagulant dispensation rates and the frequency of thromboembolic or bleeding events in people with NVAF are unknown. Rural patients have higher cardiovascular mortality and resource use than their urban counterparts [5–6]. Prescription of anticoagulants requires the ability to weigh baseline risks and, if warfarin is used, manage the inconvenience of INR monitoring [7]. These issues may be larger barriers in rural areas due to less specialist physician and laboratory access. Direct oral anticoagulants (DOACs), such as dabigatran [8], rivaroxaban [9], apixaban [10], and edoxaban [11] are at least as effective for preventing stroke and systemic embolism, are associated with fewer bleeding complications in randomized trials, and do not require INR monitoring for safe use. Significant differences in anticoagulant prescription and outcomes in rural versus urban NVAF patients in the pre-DOAC era might represent an argument in support of targeted interventions to enhance use of DOACs in rural areas, where newer therapies tend to disseminate more slowly [12].

This study was therefore conducted to determine if rural NVAF patients were less likely to be dispensed anticoagulation therapy and more likely to experience SSE, bleeding or mortality than urban NVAF patients in the era prior to introduction of DOACs.

## Methods

### Study population

The study cohort was developed using de-identified linked provincial administrative databases [Alberta Ambulatory Care Classification System (AACCS), Alberta Hospital Discharge Abstract Database (DAD), Statistics Canada neighbourhood socioeconomic data, Alberta Health Practitioner Claims Database (AHPC), Alberta Health Care Insurance Plan Registry, and the Alberta Blue Cross Drug Database]. The cohort is composed of adult Albertans (age ≥ 20 years) diagnosed with incident NVAF in any healthcare encounter from April 1, 1999 –December 31, 2008. Incident cases were identified by the absence of a NVAF diagnostic code in any of the databases during the washout period (minimum of 5 years for DAD database, 6 years for AHPC and 1 year for AACCS). We defined rural vs. urban status based on the second digit of the patient’s home address postal code. All geographical areas within Canada are included in the postal code system and no populations were excluded from this analysis. This method has been described by Statistics Canada to identify rural populations in Canada [13] and has been employed in other Canadian based administrative database studies [6]. This study was approved by the University of Alberta Health Research Ethics Board (Pro00040049).

### Defining NVAF

NVAF was defined using ICD-9-CM code 427.3x or ICD-10 code I48.x in any diagnosis field in any one of the databases. These codes have a specificity of 95–99.9%, positive predictive value (PPV) 91–99%, and sensitivity reported to be 95% in one study that used inpatient, outpatient, and emergency department (ED) data akin to what we accessed in Alberta [14–15]. To minimize misclassification of transient single episodes of AF as chronic NVAF, 2 diagnoses were required at separate healthcare encounters more than 30 days apart within the first year of diagnosis to meet case definition [16]. Patients were excluded if they had valvular AF [17].

### Comorbidities

Baseline comorbidities were defined as conditions present at the time of NVAF diagnosis (at index visit and all healthcare encounters within preceding 12 months). The ICD-9-CM and 10 codes for comorbidities have been previously validated in the Alberta databases [15, 18–20] (available from authors on request).

### Determination of SSE risk

Three validated stroke prediction scores, CHADS_2_ [21], CHA_2_DS_2_-VASc [22], R_2_CHADS_2_ [23], were calculated for each patient based on comorbidity profiles at the NVAF index date.

### Determination of bleeding risk

ATRIA [24], HASBLED [25] and HEMORR_2_HAGES [26] scores were calculated. Modifications to scores for information not available in administrative databases (such as labile INR and genetic factors) were applied as per prior studies [27–30].

### Medication use

Medication information was obtained by linking the databases with drug dispensation information from Alberta Blue Cross for patients ≥ 65 years old. Warfarin users were defined as those who filled a warfarin prescription in the 90 days prior to the incident NVAF diagnosis or within the subsequent 365 days. Aspirin use was not accurately determined as it can be obtained over the counter without a prescription.

### Outcomes

An SSE event was defined as a stroke or systemic embolism occurring after the index NVAF date using associated ICD-9-CM and ICD-10 codes. A bleeding event was defined as any intracranial hemorrhage, gastrointestinal bleeding, genitourinary bleeding, or respiratory tract bleeding using the ICD-9-CM and ICD-10 codes (available from authors on request). These codes have a sensitivity of 93%, specificity of 88%, and PPV of 91% [30–33].

Secondary outcomes included hospitalizations, ED visits, outpatient physician office visits, any bleeding, and a composite of all cause mortality or SSE in the first year after incident NVAF diagnosis.

### Statistical analysis

Baseline characteristics were displayed as frequencies (%), means (SD) or medians (IQR) and compared using χ^2^tests, t-tests or Wilcoxin rank sum tests, respectively. Warfarin dispensation was compared using the Cochran-Mantel-Haenszel test for association, controlling for levels of risk scores. One year outcomes were compared using adjusted odds ratios (aOR) from multivariate logistic regression models. All multivariate logistic models included urban/rural status, patient age, gender, year of index diagnosis, location of index diagnosis (hospital vs. ED vs. outpatient office) as well as other statistically significant covariates including health system utilization and comorbidities, as determined by stepwise variable selection with entry criteria of 0.10. The Hosmer-Lemeshow goodness of fit test was used to validate calibration for each model. Cox proportional hazards models were used to analyze one year outcomes in the elderly population to confirm results from the logistic models as well as to assess the impact of warfarin usage. These models included a time-varying binary covariate for warfarin prescriptions such that patients without a prescription were coded as non-warfarin until the time at which they received a prescription. All tests were two sided with significance level equal to 0.05. Statistical analyses were performed using SAS v9.3 (Cary, NC).

## Results

**Fig 1:**
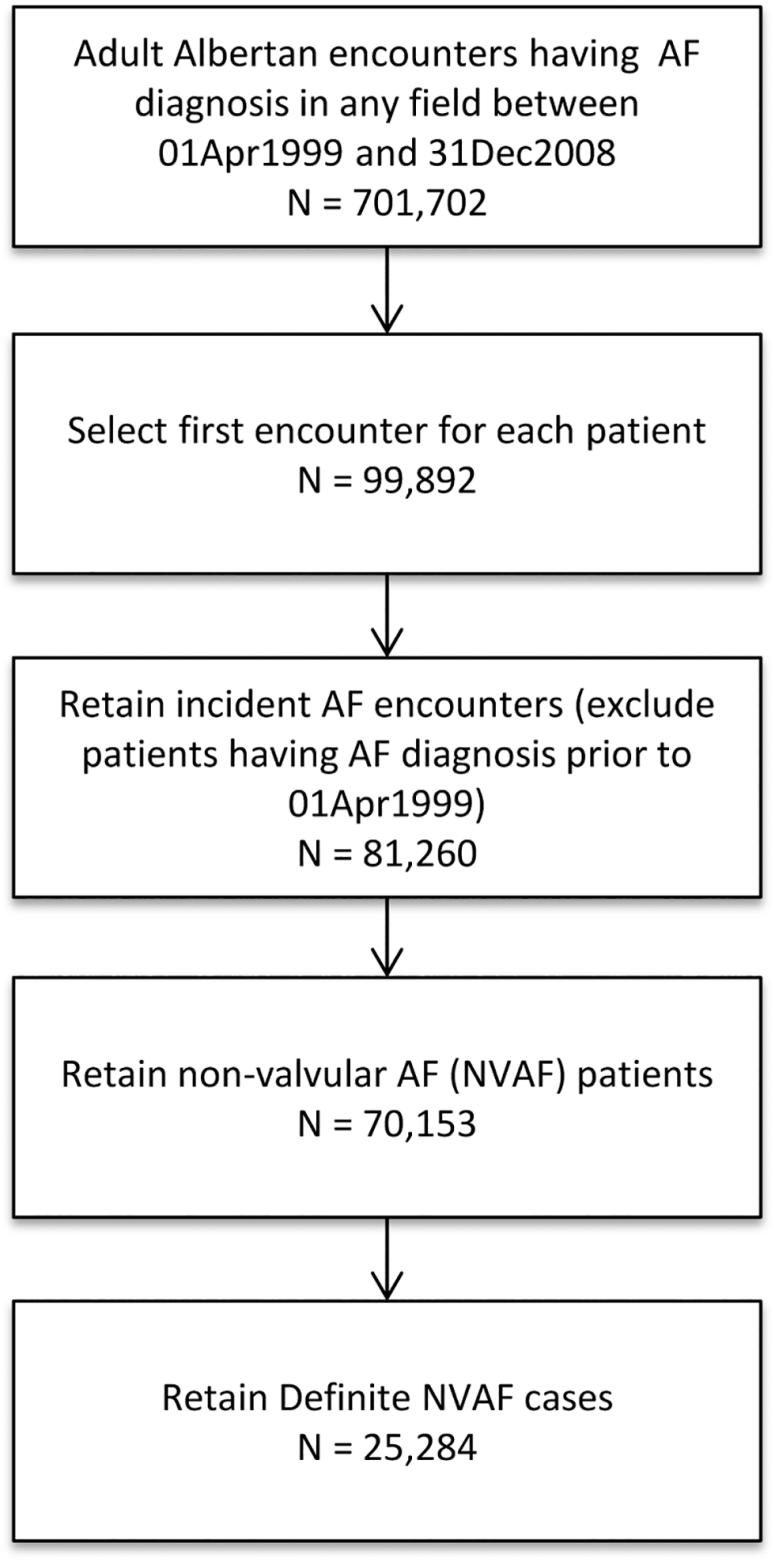
Derivation of Cohort.

A total of 25,284 were diagnosed with NVAF April 1, 1999 and December 31, 2008 (Fig 1). 20,239 (80%) were urban patients and 5045 (20%) were rural patients. These proportions are consistent with urban vs. rural total Albertan populations as reported by Statistics Canada (http://www.statcan.gc.ca/tables-tableaux/sum-som/l01/cst01/demo62j-eng.htm) over this 10 year period. Rural patients were slightly older (mean 70.9 versus 70.2 years, p = 0.001), more likely to be male, and exhibited higher comorbidity burdens at the time of diagnosis. Baseline prevalence of prior cerebrovascular disease was similar in both groups (8.7% vs. 9.1%, p = 0.41). Although rural patients exhibited higher baseline risk for SSE, urban patients exhibited higher baseline bleeding risk (Table 1), with no appreciable differences in these patterns over the years studied (S1 Table). Rural patients were more likely to be diagnosed in a hospital setting and had fewer outpatient physician visits but more hospitalizations and ED visits in the year preceding their NVAF diagnosis than urban patients (all p < 0.0001, Table 1; baseline characteristics of subgroup age ≥65 found in S2 Table).

**Table 1:** Baseline Characteristics of N = 25,284 Rural and Urban Residents with incident non-valvular atrial fibrillation (NVAF).

	NVAF		
Characteristics	Rural	Urban	P-value
**No. of patients**	5045	20239	
**Mean (SD) age, years**	70.9 (13.1)	70.2 (14.0)	0.0014
**Age > = 65 years**	3675 (72.8)	14264 (70.5)	0.0009
**Female**	2104 (41.7)	9058 (44.8)	< .0001
**Neighborhood Household Income Quintile**			
**Missing**	359 (7.1)	814 (4.0)	< .0001
**1 (lowest)**	926 (18.4)	3754 (18.5)	
**2**	1487 (29.5)	3273 (16.2)	
**3**	1350 (26.8)	3567 (17.6)	
**4**	691 (13.7)	4191 (20.7)	
**5 (highest)**	232 (4.6)	4640 (22.9)	
**Aboriginal**	181 (3.6)	185 (0.9)	< .0001
**Location of initial diagnosis**			
**Hospital**	2254 (44.7)	7027 (34.7)	< .0001
**ED** ^**a**^	964 (19.1)	5012 (24.8)	
**Ambulatory/Office Setting**	1827 (36.2)	8200 (40.5)	
**Median (IQR) no. of office-based physician visits in year prior to diagnosis**	7 (4, 12)	9 (5, 15)	< .0001
**No. of ED** ^**a**^ **visits in year prior to diagnosis**			
**0**	2354 (46.7)	12487 (61.7)	< .0001
**1**	1037 (20.6)	3815 (18.8)	
**2–4**	1090 (21.6)	3054 (15.1)	
**5+**	564 (11.2)	883 (4.4)	
**No. of hospitalizations in year prior to diagnosis**			
**0**	3863 (76.6)	16558 (81.8)	< .0001
**1**	714 (14.2)	2543 (12.6)	
**2+**	468 (9.3)	1138 (5.6)	
**Comorbidities:**			
**Ischemic Heart Disease**	1358 (26.9)	5541 (27.4)	0.51
**Diabetes**	939 (18.6)	3353 (16.6)	0.0005
**Heart Failure**	1167 (23.1)	3778 (18.7)	< .0001
**Cerebrovascular disease**	441 (8.7)	1845 (9.1)	0.41
**Ischemic Stroke**	112 (2.2)	601 (3.0)	0.004
**TIA** ^**a**^	202 (4.0)	716 (3.5)	0.11
**Intracranial Hemorrhage**	29 (0.6)	107 (0.5)	0.69
**Systemic Embolism**	25 (0.5)	146 (0.7)	0.080
**GI Bleeding**	162 (3.2)	663 (3.3)	0.82
**Hypertension**	2625 (52.0)	10645 (52.6)	0.47
**Peripheral Vascular Disease**	258 (5.1)	1123 (5.5)	0.22
**Chronic Pulmonary Disease**	1116 (22.1)	3936 (19.4)	< .0001
**Cancer**	531 (10.5)	2411 (11.9)	0.006
**Dementia**	164 (3.3)	765 (3.8)	0.074
**Peptic Ulcer Disease**	131 (2.6)	469 (2.3)	0.24
**Chronic Kidney Disease**	326 (6.5)	1461 (7.2)	0.061
**Abnormal liver function test**	51 (1.0)	283 (1.4)	0.031
**Anemia**	478 (9.5)	2221 (11.0)	0.002
**Low platelet count**	43 (0.9)	186 (0.9)	0.65
**Excessive falls**	336 (6.7)	1328 (6.6)	0.81
**Alcohol related diagnoses**	133 (2.6)	508 (2.5)	0.61
**Stroke and bleeding risk scores**			
**CHADS2 > = 2**	2326 (46.1)	9046 (44.7)	0.072
**CHADS2-VASC > = 2**	3821 (75.7)	14967 (74.0)	0.0093
**R2CHADS2 > = 2**	2402 (47.6)	9394 (46.4)	0.13
**ATRIA > = 4**	542 (10.7)	2385 (11.8)	0.039
**HASBLED > = 3**	540 (10.7)	2317 (11.4)	0.14
**HEMORR2HAGES > = 2**	2149 (42.6)	9003 (44.5)	0.016
**Office-based physician visit in year prior to diagnosis**	4685 (92.9)	19163 (94.7)	< .0001
**GP** ^**a**^	4620 (91.6)	18803 (92.9)	0.001
**Specialist, Internal Medicine/Cardiology**	1348 (26.7)	9341 (46.2)	< .0001

Table 1 legend

^a^ ED represents emergency department; TIA represents transient ischemic attack; GP represents general practitioner.

Numbers are n (%) unless otherwise specified. P-values are calculated using Chi-Square test (proportion), t-test (mean), or Wilcoxin rank sum test (median)

Most subjects were ≥65 years old. Baseline cardiovascular medications were similar in both groups (S3 Table) and as expected none of the cohort filled prescriptions for dabigatran, rivaroxaban, apixaban or edoxaban.

Within 90 days of new NVAF diagnosis, 66.1% of rural patients had filled a prescription for warfarin compared to 65.7% of urban patients (p = 0.65). By 365 days, warfarin dispensation rates had increased to 72.9% vs. 72.9% (p = 0.98). Controlling for SSE or bleeding risk, there was no statistically significant association between warfarin dispensation and rural/urban residence (Table 2). While warfarin use did not vary significantly across SSE risk categories for either group, both rural and urban patients in higher bleeding risk categories were less likely to be using warfarin at 90 and 365 days (Table 2).

**Table 2:** Incident non-valvular atrial fibrillation (NVAF) Cohort, Age 65+ (N = 17939): OAC^a^ prescription by urban/rural residence.

Score	Patient Count	OAC^a^ prescription in 90 days prior or 90 days post AF^a^ diagnosis			OAC^a^ prescription in 90 days prior or 365 days post AF^a^ diagnosis		
		Rural (n = 3,675)	Urban (n = 14,264)	p-value	Rural (n = 3,675)	Urban (n = 14,264)	p-value
**CHADS**_**2**_							
**0,1**	7695	1052 (66.1)	3932 (64.4)		1176 (73.9)	4429 (72.6)	
**≥2**	10244	1378 (66.1)	5443 (66.7)	0.64	1503 (72.1)	5972 (73.2)	0.98
**CHA**_**2**_ **DS**_**2**_ **-VASC**							
**0,1**	1205	189 (66.1)	590 (64.2)		218 (76.2)	671 (73.0)	
**> = 2**	16734	2241 (66.1)	8785 (65.8)	0.64	2461 (72.6)	9730 (72.9)	0.97
**R**_**2**_ **CHADS**_**2**_							
**0,1**	7414	1020 (66.3)	3799 (64.6)		1138 (74.0)	4285 (72.9)	
**> = 2**	10525	1410 (66.0)	5576 (66.5)	0.64	1541 (72.1)	6116 (72.9)	0.98
**ATRIA**							
**0,1,2,3**	15269	2155 (67.7)	8161 (67.5)		2371 (74.5)	9040 (74.8)	
**> = 4**	2670	275 (56.0)	1214 (55.7)	0.84	308 (62.7)	1361 (62.5)	0.76
**HASBLED** ^**b**^							
**0,1,2**	15169	2093 (66.5)	7975 (66.3)		2305 (73.2)	8858 (73.7)	
**> = 3**	2770	337 (64.1)	1400 (62.4)	0.69	374 (71.1)	1543 (68.8)	0.92
**HEMORR**_**2**_ **HAGES**							
**0,1**	7718	1164 (68.8)	4069 (67.5)		1293 (76.4)	4551 (75.5)	
**> = 2**	10221	1266 (63.9)	5306 (64.4)	0.76	1386 (69.9)	5850 (71.0)	0.80

Table 2 legend

^**a**^ AF represents atrial fibrillation; OAC represents Oral Anticoagulant.

^**b**^ HASBLED does not take into account aspirin intake

Numbers are n (%) except for patient count and p-value.

P-values are from Cochran-Mantel-Haenszel test for association between urban/rural and OAC prescription, controlling for risk scores (two levels as specified)

Within one year of NVAF diagnosis, 7.8% of patients died, 3.9% suffered a SSE event, and 5.9% had at least one bleeding event, with no significant differences between rural and urban patients even after multivariate adjustment (aOR for rural vs. urban patients 1.06 [0.95–1.28] for death or SSE and 0.93 [0.81–1.06] for bleeding, S4 Table). Consistent with pre-diagnosis patterns, urban patients were less likely to be hospitalized (38.1% vs. 46.5%, aOR 0.82, 95%CI 0.77–0.89) or to visit an ED (62.7% vs. 76.4%, aOR 0.61, 95%CI 0.56–0.66). Both urban and rural patients exhibited similar rates of outpatient office visits (98.5% vs. 98.3%, aOR 0.84, 95%CI 0.64–1.10). In our Cox-proportional hazards model in which we treated warfarin dispensation as a time-varying covariate, warfarin users at 1 year were at lower risk of death (7.0% vs 18.1%, aHR 0.60, 95% CI 0.54–0.66), stroke/TIA/systemic embolism (4.6% vs 5.5%, aHR 0.61, 95% CI 0.53–0.71), or all-cause hospitalization (43.1% vs 53.4%, aHR 0.77, 95% CI 0.74–0.81) but at higher risk of bleeding (7.5% vs 6.2%, aHR 1.51, 95% CI 1.33–1.72) (S4 Table).

## Discussion

In this large population-based cohort of patients with incident NVAF, we found no difference between rates of warfarin dispensation, mortality, SSE, or bleeding in urban vs. rural NVAF patients. Consistent with prior literature, our data show a risk-treatment paradox in NVAF, with underuse of anticoagulation in high baseline SSE risk patients [17]. Despite the higher bleeding rates, all-cause mortality, SSE and all-cause hospitalization were significantly lower in patients dispensed warfarin. Our results suggest that bleeding risk estimation may have a larger influence on clinical decisions for NVAF patients than SSE risk estimation since warfarin dispensation rates did not vary by SSE risk but those with higher bleeding risk were less likely to be dispensed warfarin. This is consistent with results of published physician surveys [34]. Though we did not find treatment differences between urban and rural NVAF patients that would justify targeted use of DOAC drugs, the underuse of anticoagulant drugs in both urban and rural patients with high SSE risk and the sensitivity of prescribers to bleeding risk represent an opportunity for expanded use of DOAC drugs, which had favourable bleeding profiles compared with warfarin in randomized trials.

Similar to the findings in our NVAF population, prior studies in patients with other cardiovascular disease have also found no difference in all-cause mortality between urban and rural patients with HF despite the fact that urban patients had fewer hospitalizations or ED visits after diagnosis [6]. Others have reported better access to specialized care for urban patients with HF, coronary artery disease, or diabetes compared to their rural counterparts, with resultant lower mortality and rates of hospitalization or ED visits for urban individuals [35–39]. A recently published Canadian study from the province of Quebec [40] also found no difference in major thrombotic and bleeding outcomes between rural and urban atrial fibrillation patients and a general underuse of warfarin. Unlike our findings, there was a small but statistically significant increased rate of warfarin prescriptions in rural patients as well as a trend to increased warfarin prescriptions in patients with a higher CHADS2 score. The Quebec study selected atrial fibrillation patients via a hospital discharge database prior to linking to the physician and prescription claims database and used one atrial fibrillation code to define incident cases. Warfarin prescriptions were only tracked within the first 7 days of discharge. Average age, CHADS2 and HASBLED scores suggest a more selected higher risk population of AF patients. Our study captures a more broad population of atrial fibrillation patients with a longer term dispensation pattern of warfarin which may account for some of noted differences.

There are several limitations of our study. Data was collected retrospectively from administrative databases, and residual confounding may remain despite careful adjustment. Case definitions and comorbidity codes were previously validated in Alberta administrative databases, minimizing misclassification bias [32, 34]. Laboratory data linkage was not available, resulting in no information on time in therapeutic range and thus it was not possible to compare the adequacy of warfarin anticoagulation for rural vs. urban patients. The fact that there were no significant differences in rates of SSE or bleeding suggests that INR control was likely similar. Although the rural cohort appeared to have a higher comorbidity burden at baseline, this may have been artifactual and a reflection of the fact that more of the rural cohort were hospitalized at the time of their initial NVAF diagnosis or in the preceding year; the discharge abstract database captures up to 25 diagnoses per patient while the outpatient physician billing date only captures up to 3 diagnoses. Regardless, our data likely underestimated comorbidity burden for both rural and urban patients since administrative codes have excellent specificity but poorer sensitivity for several elements of the CHADS derivative scores. The advantage of our dataset is that it captures all physician-entered diagnoses and thus accurately captures the comorbidity burden recognized by treating physicians. As ASA can be obtained over the counter, its use was under-captured in our study and we cannot evaluate whether ASA use differed between rural and urban patients and whether this impacted SSE or bleeding rates. Finally, our study was conducted in a publically funded, universal access Canadian health care system, and it is possible that rural-urban differences may be more apparent in systems where financial barriers impact health care access.

## Conclusion

Despite reduced access to outpatient resources in rural communities and subsequent downstream impact on health service use with increased hospitalizations and ED visits, we found no differences in warfarin use or clinical outcomes between rural and urban patients with NVAF. Warfarin appeared to be under-used in patients at high SSE risk regardless of place of residence. Future efforts should be directed at addressing the risk-treatment paradox for all NVAF patients.

## Supporting Information

S1 Table Incident NVAF Cohort, Age 65+ (N = 17939): OAC prescription by urban/rural residence. (DOCX)

S2 Table Baseline Characteristics of N = 25,284 Rural and Urban Residents with Incident Definite Non-Valvular Atrial Fibrillation (NVAF), age ≥65 years subgroup (DOCX)

S3 Table Medication dispensation patterns. (DOCX)

S4 Table Crude and adjusted odds ratios for 1-year outcomes of urban residents compared to rural residents with incident non-valvular atrial fibrillation. (DOCX)
