# Supplementary material for: Impact of Rural Residence on Warfarin Use and Clinical Events in Patients with Non-Valvular Atrial Fibrillation: A Canadian Population Based Study
Source: PLoS One. 2015 Oct 14;10(10):e0140607. doi: 10.1371/journal.pone.0140607 (PMC4605516; doi:10.1371/journal.pone.0140607)
Supplement: S1 Table — (DOCX) [file pone.0140607.s001.docx]

**S1 Table. Trend of Risk Scores for 25,284 patients with incident non-valvular atrial fibrillation (NVAF), by year**

|  |  |  |  |  |  | **Year** |  |  |  |  |  |  |
| --- | --- | --- | --- | --- | --- | --- | --- | --- | --- | --- | --- | --- |
| **Characteristics** | **1999** | **2000** | **2001** | **2002** | **2003** | **2004** | **2005** | **2006** | **2007** | **2008** | **Total** | **P-value** |
| **No. of patients** | 1430 | 1830 | 1925 | 2053 | 2314 | 2881 | 3136 | 3205 | 3207 | 3303 | 25284 |  |
| **Location of initial diagnosis** |  |  |  |  |  |  |  |  |  |  |  |  |
| **Hospital** | 685 (47.9) | 829 (45.3) | 851 (44.2) | 826 (40.2) | 960 (41.5) | 960 (33.3) | 1138 (36.3) | 1004 (31.3) | 1022 (31.9) | 1006 (30.5) | 9281 (36.7) | <.0001 |
| **ED** | 294 (20.6) | 406 (22.2) | 450 (23.4) | 550 (26.8) | 554 (23.9) | 652 (22.6) | 688 (21.9) | 811 (25.3) | 733 (22.9) | 838 (25.4) | 5976 (23.6) |  |
| **Ambulatory/Office Setting** | 451 (31.5) | 595 (32.5) | 624 (32.4) | 677 (33.0) | 800 (34.6) | 1269 (44.0) | 1310 (41.8) | 1390 (43.4) | 1452 (45.3) | 1459 (44.2) | 10027 (39.7) |  |
|  |  |  |  |  |  |  |  |  |  |  |  |  |
| **CHADS2 >= 2** | 634 (44.3) | 804 (43.9) | 850 (44.2) | 880 (42.9) | 1043 (45.1) | 1292 (44.8) | 1476 (47.1) | 1421 (44.3) | 1459 (45.5) | 1513 (45.8) | 11372 (45.0) | 0.043 |
| **CHADS2-VASC >= 2** | 1101 (77.0) | 1386 (75.7) | 1430 (74.3) | 1537 (74.9) | 1711 (73.9) | 2145 (74.5) | 2313 (73.8) | 2402 (74.9) | 2364 (73.7) | 2399 (72.6) | 18788 (74.3) | 0.003 |
| **R2CHADS2 >= 2** | 651 (45.5) | 824 (45.0) | 875 (45.5) | 911 (44.4) | 1087 (47.0) | 1337 (46.4) | 1534 (48.9) | 1480 (46.2) | 1528 (47.6) | 1569 (47.5) | 11796 (46.7) | 0.0054 |
| **ATRIA >= 4** | 181 (12.7) | 229 (12.5) | 233 (12.1) | 260 (12.7) | 289 (12.5) | 298 (10.3) | 366 (11.7) | 347 (10.8) | 357 (11.1) | 367 (11.1) | 2927 (11.6) | 0.0049 |
| **HASBLED >= 3** | 154 (10.8) | 194 (10.6) | 184 (9.6) | 231 (11.3) | 275 (11.9) | 332 (11.5) | 369 (11.8) | 341 (10.6) | 351 (10.9) | 426 (12.9) | 2857 (11.3) | 0.018 |
| **HEMORR2HAGES >= 2** | 628 (43.9) | 801 (43.8) | 822 (42.7) | 843 (41.1) | 1032 (44.6) | 1259 (43.7) | 1425 (45.4) | 1404 (43.8) | 1462 (45.6) | 1476 (44.7) | 11152 (44.1) | 0.028 |
|  |  |  |  |  |  |  |  |  |  |  |  |  |
| **CHADS2 Score** |  |  |  |  |  |  |  |  |  |  |  |  |
| **0** | 338 (23.6) | 446 (24.4) | 482 (25.0) | 514 (25.0) | 555 (24.0) | 701 (24.3) | 760 (24.2) | 785 (24.5) | 751 (23.4) | 828 (25.1) | 6160 (24.4) | 0.11 |
| **1** | 458 (32.0) | 580 (31.7) | 593 (30.8) | 659 (32.1) | 716 (30.9) | 888 (30.8) | 900 (28.7) | 999 (31.2) | 997 (31.1) | 962 (29.1) | 7752 (30.7) |  |
| **2** | 389 (27.2) | 457 (25.0) | 521 (27.1) | 529 (25.8) | 590 (25.5) | 787 (27.3) | 872 (27.8) | 852 (26.6) | 829 (25.8) | 853 (25.8) | 6679 (26.4) |  |
| **3** | 164 (11.5) | 238 (13.0) | 236 (12.3) | 238 (11.6) | 289 (12.5) | 315 (10.9) | 402 (12.8) | 386 (12.0) | 435 (13.6) | 428 (13.0) | 3131 (12.4) |  |
| **4** | 53 (3.7) | 80 (4.4) | 70 (3.6) | 87 (4.2) | 129 (5.6) | 150 (5.2) | 158 (5.0) | 151 (4.7) | 153 (4.8) | 185 (5.6) | 1216 (4.8) |  |
| **5** | 24 (1.7) | 27 (1.5) | 18 (0.9) | 23 (1.1) | 30 (1.3) | 34 (1.2) | 37 (1.2) | 28 (0.9) | 32 (1.0) | 41 (1.2) | 294 (1.2) |  |
| **6** | 4 (0.3) | 2 (0.1) | 5 (0.3) | 3 (0.1) | 5 (0.2) | 6 (0.2) | 7 (0.2) | 4 (0.1) | 10 (0.3) | 6 (0.2) | 52 (0.2) |  |
|  |  |  |  |  |  |  |  |  |  |  |  |  |
| **CHADS2-VASC Score** |  |  |  |  |  |  |  |  |  |  |  |  |
| **0** | 128 (9.0) | 174 (9.5) | 193 (10.0) | 206 (10.0) | 232 (10.0) | 304 (10.6) | 334 (10.7) | 346 (10.8) | 346 (10.8) | 370 (11.2) | 2633 (10.4) | 0.097 |
| **1** | 201 (14.1) | 270 (14.8) | 302 (15.7) | 310 (15.1) | 371 (16.0) | 432 (15.0) | 489 (15.6) | 457 (14.3) | 497 (15.5) | 534 (16.2) | 3863 (15.3) |  |
| **2** | 281 (19.7) | 347 (19.0) | 381 (19.8) | 424 (20.7) | 399 (17.2) | 544 (18.9) | 546 (17.4) | 644 (20.1) | 590 (18.4) | 574 (17.4) | 4730 (18.7) |  |
| **3** | 324 (22.7) | 405 (22.1) | 410 (21.3) | 471 (22.9) | 531 (22.9) | 650 (22.6) | 670 (21.4) | 727 (22.7) | 680 (21.2) | 705 (21.3) | 5573 (22.0) |  |
| **4** | 275 (19.2) | 333 (18.2) | 337 (17.5) | 351 (17.1) | 403 (17.4) | 519 (18.0) | 605 (19.3) | 552 (17.2) | 598 (18.6) | 602 (18.2) | 4575 (18.1) |  |
| **5** | 136 (9.5) | 185 (10.1) | 192 (10.0) | 179 (8.7) | 219 (9.5) | 254 (8.8) | 303 (9.7) | 306 (9.5) | 314 (9.8) | 290 (8.8) | 2378 (9.4) |  |
| **6** | 49 (3.4) | 88 (4.8) | 85 (4.4) | 80 (3.9) | 114 (4.9) | 128 (4.4) | 143 (4.6) | 132 (4.1) | 135 (4.2) | 175 (5.3) | 1129 (4.5) |  |
| **7** | 30 (2.1) | 19 (1.0) | 16 (0.8) | 25 (1.2) | 36 (1.6) | 40 (1.4) | 33 (1.1) | 34 (1.1) | 39 (1.2) | 44 (1.3) | 316 (1.2) |  |
| **8** | 5 (0.3) | 7 (0.4) | 8 (0.4) | 7 (0.3) | 9 (0.4) | 7 (0.2) | 12 (0.4) | 5 (0.2) | 8 (0.2) | 9 (0.3) | 77 (0.3) |  |
| **9** | 1 (0.1) | 2 (0.1) | 1 (0.1) | 0 (0.0) | 0 (0.0) | 3 (0.1) | 1 (0.0) | 2 (0.1) | 0 (0.0) | 0 (0.0) | 10 (0.0) |  |
|  |  |  |  |  |  |  |  |  |  |  |  |  |
| **R2CHADS2 Score** |  |  |  |  |  |  |  |  |  |  |  |  |
| **0** | 332 (23.2) | 436 (23.8) | 476 (24.7) | 510 (24.8) | 545 (23.6) | 684 (23.7) | 746 (23.8) | 769 (24.0) | 734 (22.9) | 812 (24.6) | 6044 (23.9) | 0.076 |
| **1** | 447 (31.3) | 570 (31.1) | 574 (29.8) | 632 (30.8) | 682 (29.5) | 860 (29.9) | 856 (27.3) | 956 (29.8) | 945 (29.5) | 922 (27.9) | 7444 (29.4) |  |
| **2** | 371 (25.9) | 435 (23.8) | 496 (25.8) | 492 (24.0) | 551 (23.8) | 728 (25.3) | 809 (25.8) | 797 (24.9) | 763 (23.8) | 806 (24.4) | 6248 (24.7) |  |
| **3** | 153 (10.7) | 209 (11.4) | 219 (11.4) | 241 (11.7) | 274 (11.8) | 278 (9.6) | 380 (12.1) | 357 (11.1) | 418 (13.0) | 380 (11.5) | 2909 (11.5) |  |
| **4** | 67 (4.7) | 96 (5.2) | 93 (4.8) | 116 (5.7) | 157 (6.8) | 201 (7.0) | 205 (6.5) | 190 (5.9) | 207 (6.5) | 212 (6.4) | 1544 (6.1) |  |
| **5** | 42 (2.9) | 63 (3.4) | 50 (2.6) | 43 (2.1) | 74 (3.2) | 94 (3.3) | 93 (3.0) | 95 (3.0) | 94 (2.9) | 122 (3.7) | 770 (3.0) |  |
| **6** | 13 (0.9) | 18 (1.0) | 11 (0.6) | 14 (0.7) | 25 (1.1) | 29 (1.0) | 36 (1.1) | 34 (1.1) | 38 (1.2) | 40 (1.2) | 258 (1.0) |  |
| **7** | 4 (0.3) | 3 (0.2) | 4 (0.2) | 4 (0.2) | 5 (0.2) | 5 (0.2) | 10 (0.3) | 5 (0.2) | 7 (0.2) | 7 (0.2) | 54 (0.2) |  |
| **8** | 1 (0.1) | 0 (0.0) | 2 (0.1) | 1 (0.0) | 1 (0.0) | 2 (0.1) | 1 (0.0) | 2 (0.1) | 1 (0.0) | 2 (0.1) | 13 (0.1) |  |
|  |  |  |  |  |  |  |  |  |  |  |  |  |
| **ATRIA Score** |  |  |  |  |  |  |  |  |  |  |  |  |
| **0** | 376 (26.3) | 506 (27.7) | 565 (29.4) | 596 (29.0) | 612 (26.4) | 786 (27.3) | 880 (28.1) | 869 (27.1) | 879 (27.4) | 935 (28.3) | 7004 (27.7) | <.0001 |
| **1** | 295 (20.6) | 375 (20.5) | 400 (20.8) | 478 (23.3) | 525 (22.7) | 664 (23.0) | 695 (22.2) | 761 (23.7) | 744 (23.2) | 770 (23.3) | 5707 (22.6) |  |
| **2** | 253 (17.7) | 320 (17.5) | 316 (16.4) | 321 (15.6) | 347 (15.0) | 462 (16.0) | 436 (13.9) | 479 (14.9) | 463 (14.4) | 447 (13.5) | 3844 (15.2) |  |
| **3** | 325 (22.7) | 400 (21.9) | 411 (21.4) | 398 (19.4) | 541 (23.4) | 671 (23.3) | 759 (24.2) | 749 (23.4) | 764 (23.8) | 784 (23.7) | 5802 (22.9) |  |
| **4** | 60 (4.2) | 71 (3.9) | 77 (4.0) | 88 (4.3) | 103 (4.5) | 108 (3.7) | 142 (4.5) | 115 (3.6) | 122 (3.8) | 137 (4.1) | 1023 (4.0) |  |
| **5** | 40 (2.8) | 48 (2.6) | 50 (2.6) | 49 (2.4) | 59 (2.5) | 54 (1.9) | 72 (2.3) | 64 (2.0) | 72 (2.2) | 56 (1.7) | 564 (2.2) |  |
| **6** | 58 (4.1) | 72 (3.9) | 68 (3.5) | 92 (4.5) | 98 (4.2) | 109 (3.8) | 127 (4.0) | 130 (4.1) | 125 (3.9) | 126 (3.8) | 1005 (4.0) |  |
| **7** | 15 (1.0) | 30 (1.6) | 21 (1.1) | 23 (1.1) | 24 (1.0) | 25 (0.9) | 20 (0.6) | 34 (1.1) | 30 (0.9) | 43 (1.3) | 265 (1.0) |  |
| **8** | 0 (0.0) | 1 (0.1) | 2 (0.1) | 3 (0.1) | 2 (0.1) | 2 (0.1) | 1 (0.0) | 0 (0.0) | 3 (0.1) | 2 (0.1) | 16 (0.1) |  |
| **9** | 6 (0.4) | 4 (0.2) | 11 (0.6) | 4 (0.2) | 3 (0.1) | 0 (0.0) | 3 (0.1) | 2 (0.1) | 4 (0.1) | 3 (0.1) | 40 (0.2) |  |
| **10** | 2 (0.1) | 3 (0.2) | 4 (0.2) | 1 (0.0) | 0 (0.0) | 0 (0.0) | 1 (0.0) | 2 (0.1) | 1 (0.0) | 0 (0.0) | 14 (0.1) |  |
|  |  |  |  |  |  |  |  |  |  |  |  |  |
| **HASBLED Score** |  |  |  |  |  |  |  |  |  |  |  |  |
| **0** | 214 (15.0) | 285 (15.6) | 330 (17.1) | 328 (16.0) | 375 (16.2) | 453 (15.7) | 547 (17.4) | 544 (17.0) | 599 (18.7) | 615 (18.6) | 4290 (17.0) | <.0001 |
| **1** | 511 (35.7) | 679 (37.1) | 710 (36.9) | 743 (36.2) | 813 (35.1) | 1021 (35.4) | 1007 (32.1) | 1099 (34.3) | 1031 (32.1) | 1067 (32.3) | 8681 (34.3) |  |
| **2** | 551 (38.5) | 672 (36.7) | 701 (36.4) | 751 (36.6) | 851 (36.8) | 1075 (37.3) | 1213 (38.7) | 1221 (38.1) | 1226 (38.2) | 1195 (36.2) | 9456 (37.4) |  |
| **3** | 133 (9.3) | 155 (8.5) | 150 (7.8) | 191 (9.3) | 231 (10.0) | 277 (9.6) | 314 (10.0) | 278 (8.7) | 291 (9.1) | 358 (10.8) | 2378 (9.4) |  |
| **4** | 19 (1.3) | 35 (1.9) | 25 (1.3) | 33 (1.6) | 42 (1.8) | 50 (1.7) | 47 (1.5) | 61 (1.9) | 60 (1.9) | 65 (2.0) | 437 (1.7) |  |
| **5** | 2 (0.1) | 4 (0.2) | 8 (0.4) | 7 (0.3) | 2 (0.1) | 4 (0.1) | 8 (0.3) | 2 (0.1) | 0 (0.0) | 3 (0.1) | 40 (0.2) |  |
| **6** | 0 (0.0) | 0 (0.0) | 0 (0.0) | 0 (0.0) | 0 (0.0) | 1 (0.0) | 0 (0.0) | 0 (0.0) | 0 (0.0) | 0 (0.0) | 1 (0.0) |  |
| **7** | 0 (0.0) | 0 (0.0) | 1 (0.1) | 0 (0.0) | 0 (0.0) | 0 (0.0) | 0 (0.0) | 0 (0.0) | 0 (0.0) | 0 (0.0) | 1 (0.0) |  |
|  |  |  |  |  |  |  |  |  |  |  |  |  |
| **HEMORR2HAGES Score** |  |  |  |  |  |  |  |  |  |  |  |  |
| **0** | 327 (22.9) | 430 (23.5) | 493 (25.6) | 500 (24.4) | 541 (23.4) | 654 (22.7) | 737 (23.5) | 741 (23.1) | 741 (23.1) | 790 (23.9) | 5954 (23.5) | 0.055 |
| **1** | 475 (33.2) | 599 (32.7) | 610 (31.7) | 710 (34.6) | 741 (32.0) | 968 (33.6) | 974 (31.1) | 1060 (33.1) | 1004 (31.3) | 1037 (31.4) | 8178 (32.3) |  |
| **2** | 379 (26.5) | 469 (25.6) | 516 (26.8) | 498 (24.3) | 623 (26.9) | 752 (26.1) | 843 (26.9) | 842 (26.3) | 904 (28.2) | 838 (25.4) | 6664 (26.4) |  |
| **3** | 171 (12.0) | 222 (12.1) | 207 (10.8) | 227 (11.1) | 275 (11.9) | 362 (12.6) | 409 (13.0) | 398 (12.4) | 388 (12.1) | 441 (13.4) | 3100 (12.3) |  |
| **4** | 59 (4.1) | 82 (4.5) | 68 (3.5) | 82 (4.0) | 104 (4.5) | 115 (4.0) | 136 (4.3) | 129 (4.0) | 123 (3.8) | 137 (4.1) | 1035 (4.1) |  |
| **5** | 12 (0.8) | 24 (1.3) | 21 (1.1) | 32 (1.6) | 29 (1.3) | 24 (0.8) | 31 (1.0) | 29 (0.9) | 34 (1.1) | 54 (1.6) | 290 (1.1) |  |
| **6** | 6 (0.4) | 4 (0.2) | 9 (0.5) | 3 (0.1) | 1 (0.0) | 5 (0.2) | 4 (0.1) | 5 (0.2) | 13 (0.4) | 5 (0.2) | 55 (0.2) |  |
| **7** | 0 (0.0) | 0 (0.0) | 1 (0.1) | 1 (0.0) | 0 (0.0) | 1 (0.0) | 1 (0.0) | 1 (0.0) | 0 (0.0) | 1 (0.0) | 6 (0.0) |  |
| **8** | 1 (0.1) | 0 (0.0) | 0 (0.0) | 0 (0.0) | 0 (0.0) | 0 (0.0) | 1 (0.0) | 0 (0.0) | 0 (0.0) | 0 (0.0) | 2 (0.0) |  |

Numbers are n (%) unless otherwise specified.

P-values for binary variables calculated using Cochran-Armitage test for trend; p-values for categorical variables calculated using Chi-Square test.
