# Supplementary material for: Impact of Rural Residence on Warfarin Use and Clinical Events in Patients with Non-Valvular Atrial Fibrillation: A Canadian Population Based Study
Source: PLoS One. 2015 Oct 14;10(10):e0140607. doi: 10.1371/journal.pone.0140607 (PMC4605516; doi:10.1371/journal.pone.0140607)
Supplement: S2 Table — (DOCX) [file pone.0140607.s002.docx]

**S2 Table. Baseline Characteristics of N = 25,284 Rural and Urban Residents with incident definite non-valvular atrial fibrillation (NVAF), age ≥65 years subgroup.**

|  | **Definite NVAF, age 65 and older** | | |
| --- | --- | --- | --- |
| **Characteristics** | **Rural** | **Urban** | **P-value** |
| **No. of patients** | 3675 | 14264 |  |
| **Mean (SD) age, years** | 77.2 (7.7) | 77.6 (7.4) | 0.0068 |
| **Age >= 65 years** | 3675 (100.0) | 14264 (100.0) | n/a |
| **Female** | 1651 (44.9) | 7238 (50.7) | <.0001 |
| **Neighborhood Household Income Quintile** |  |  |  |
| **Missing** | 267 (7.3) | 502 (3.5) | <.0001 |
| **1 (lowest)** | 681 (18.5) | 2983 (20.9) |  |
| **2** | 1099 (29.9) | 2486 (17.4) |  |
| **3** | 996 (27.1) | 2595 (18.2) |  |
| **4** | 494 (13.4) | 2873 (20.1) |  |
| **5 (highest)** | 138 (3.8) | 2825 (19.8) |  |
| **Aboriginal** | 92 (2.5) | 51 (0.4) | <.0001 |
| **Location of initial diagnosis** |  |  |  |
| **Hospital** | 1755 (47.8) | 5574 (39.1) | <.0001 |
| **ED**^a^ | 643 (17.5) | 3059 (21.4) |  |
| **Ambulatory/Office Setting** | 1277 (34.7) | 5631 (39.5) |  |
| **Median (IQR) no. of office-based physician visits in year prior to diagnosis** | 8 (4, 13) | 10 (6, 16) | <.0001 |
| **No. of ED**^a^ **visits in year prior to diagnosis** |  |  |  |
| **0** | 1665 (45.3) | 8653 (60.7) | <.0001 |
| **1** | 768 (20.9) | 2740 (19.2) |  |
| **2-4** | 818 (22.3) | 2240 (15.7) |  |
| **5+** | 424 (11.5) | 631 (4.4) |  |
| **No. of hospitalization in year prior to diagnosis** |  |  |  |
| **0** | 2736 (74.4) | 11391 (79.9) | <.0001 |
| **1** | 556 (15.1) | 1993 (14.0) |  |
| **2+** | 383 (10.4) | 880 (6.2) |  |
| **Comorbidities:** |  |  |  |
| **Ischemic Heart Disease** | 1102 (30.0) | 4344 (30.5) | 0.58 |
| **Diabetes** | 728 (19.8) | 2616 (18.3) | 0.041 |
| **Heart Failure** | 1000 (27.2) | 3111 (21.8) | <.0001 |
| **Cerebrovascular disease** | 383 (10.4) | 1599 (11.2) | 0.17 |
| **Ischemic Stroke** | 91 (2.5) | 512 (3.6) | 0.0008 |
| **TIA**^a^ | 175 (4.8) | 631 (4.4) | 0.38 |
| **Intracranial Hemorrhage** | 25 (0.7) | 81 (0.6) | 0.43 |
| **Systemic Embolism** | 21 (0.6) | 102 (0.7) | 0.35 |
| **GI Bleeding** | 141 (3.8) | 534 (3.7) | 0.79 |
| **Hypertension** | 2114 (57.5) | 8574 (60.1) | 0.0044 |
| **Peripheral Vascular Disease** | 229 (6.2) | 985 (6.9) | 0.15 |
| **PAD**^a^ | 140 (3.8) | 621 (4.4) | 0.15 |
| **Chronic Pulmonary Disease** | 892 (24.3) | 3143 (22.0) | 0.0038 |
| **Cancer** | 469 (12.8) | 2055 (14.4) | 0.011 |
| **Dementia** | 161 (4.4) | 743 (5.2) | 0.041 |
| **Peptic Ulcer Disease** | 110 (3.0) | 370 (2.6) | 0.18 |
| **Chronic Kidney Disease** | 273 (7.4) | 1223 (8.6) | 0.025 |
| **Abnormal liver function test** | 37 (1.0) | 179 (1.3) | 0.22 |
| **Anemia** | 419 (11.4) | 1889 (13.2) | 0.0029 |
| **Low platelet count** | 33 (0.9) | 135 (0.9) | 0.79 |
| **Excessive falls** | 290 (7.9) | 1140 (8.0) | 0.84 |
| **Alcohol related diagnoses** | 74 (2.0) | 263 (1.8) | 0.50 |
| **Stroke and bleeding risk scores** |  |  |  |
| **CHADS2 >= 2** | 2084 (56.7) | 8160 (57.2) | 0.59 |
| **CHADS2-VASC >= 2** | 3389 (92.2) | 13345 (93.6) | 0.0038 |
| **R2CHADS2 >= 2** | 2137 (58.1) | 8388 (58.8) | 0.47 |
| **ATRIA >= 4** | 491 (13.4) | 2179 (15.3) | 0.0036 |
| **HASBLED >= 3** | 526 (14.3) | 2244 (15.7) | 0.034 |
| **HEMORR2HAGES >= 2** | 1982 (53.9) | 8239 (57.8) | <.0001 |
| **Office-based physician visit in year prior to diagnosis** | 3483 (94.8) | 13728 (96.2) | <.0001 |
| **GP**^a^ | 3435 (93.5) | 13467 (94.4) | 0.029 |
| **Specialist, Internal Medicine/Cardiology** | 979 (26.6) | 6891 (48.3) | <.0001 |

^a^ ED represents emergency department; TIA, transient ischemic attack; PAD, peripheral artery disease; GP, general practitioner. Numbers are n (%) unless otherwise specified. P-values are calculated using Chi-Square test (proportion), t-test (mean), or Wilcoxin rank sum test (median)
