# Supplementary material for: Impact of Rural Residence on Warfarin Use and Clinical Events in Patients with Non-Valvular Atrial Fibrillation: A Canadian Population Based Study
Source: PLoS One. 2015 Oct 14;10(10):e0140607. doi: 10.1371/journal.pone.0140607 (PMC4605516; doi:10.1371/journal.pone.0140607)
Supplement: S3 Table — (DOCX) [file pone.0140607.s003.docx]

**S3 Table. Medication dispensation patterns**

|  |  |  |  |
| --- | --- | --- | --- |
| **Characteristics** | **Rural** | **Urban** | **P-value** |
| **Medications in 90 days prior to diagnosis for patients age 65+** |  |  |  |
| **No. of patients** | 9809 | 39152 |  |
| **ACE/ARB** | 3670 (37.4) | 14450 (36.9) | 0.35 |
| **Beta Blocker** | 2123 (21.6) | 9110 (23.3) | 0.0006 |
| **Calcium-channel blocker** | 673 (6.9) | 2844 (7.3) | 0.17 |
| **Amiodarone** | 69 (0.7) | 269 (0.7) | 0.86 |
| **Warfarin** | 1280 (13.0) | 5888 (15.0) | <.0001 |
| **OAC (non-Warfarin)** | 0 (0.0) | 0 (0.0) | n/a |
| **Plavix (Clopidogrel)** | 241 (2.5) | 1088 (2.8) | 0.079 |
| **ASA** | 103 (1.1) | 363 (0.9) | 0.26 |
