# Supplementary material for: Impact of Rural Residence on Warfarin Use and Clinical Events in Patients with Non-Valvular Atrial Fibrillation: A Canadian Population Based Study
Source: PLoS One. 2015 Oct 14;10(10):e0140607. doi: 10.1371/journal.pone.0140607 (PMC4605516; doi:10.1371/journal.pone.0140607)
Supplement: S4 Table — (DOCX) [file pone.0140607.s004.docx]

**S4 Table. Crude and adjusted odds ratios for 1-year outcomes of urban residents compared to rural residents with incident non-valvular atrial fibrillation**

| **1-yr Outcomes** | **No. events in rural residents (n=5,045)** | **No. events in urban residents (n=20,239)** | **Crude OR**^a^  **(95% CI** ^a^**)** | **Adjusted OR**^a^  **(95% CI** ^a^**)** | **Corrected RR** ^a^  **(95% CI** ^a^**)** |
| --- | --- | --- | --- | --- | --- |
| **All Patients** |  |  |  |  |  |
| **All-cause mortality** | 396 (7.8) | 1582 (7.8) | 0.995 (0.89, 1.12) | 1.10 (0.97, 1.25) | 1.09 (0.97, 1.23) |
| **All-cause non-elective hospitalization** | 2347 (46.5) | 7716 (38.1) | 0.71 (0.67, 0.75) | 0.82 (0.77, 0.89) | 0.90 (0.86, 0.94) |
| **Stroke/TIA/systemic embolism** | 220 (4.4) | 754 (3.7) | 0.85 (0.73, 0.99) | 0.86 (0.73, 1.00) | 0.86 (0.74, 1.00) |
| **Any Bleeding** | 333 (6.6) | 1149 (5.7) | 0.85 (0.75, 0.97) | 0.93 (0.81, 1.06) | 0.93 (0.82, 1.05) |
| **Composite of all-cause mortality or thromboembolic events** | 567 (11.2) | 2194 (10.8) | 0.96 (0.87, 1.06) | 1.06 (0.95, 1.18) | 1.05 (0.95, 1.15) |
| **Emergency department visits** | 3853 (76.4) | 12699 (62.7) | 0.52 (0.49, 0.56) | 0.61 (0.56, 0.66) | 0.87 (0.84, 0.89) |
| **Office-based physician visits** | 4960 (98.3) | 19941 (98.5) | 1.15 (0.90, 1.46) | 0.84 (0.64, 1.10) | 0.997 (0.99, 1.00) |
| **Older patients (age 65+) with any warfarin exposure in 90 days prior or 365 days post AF diagnosis** | | | | | |
| **All-cause mortality** | 184 (6.9) | 737 (7.1) | 1.03 (0.87, 1.22) | 1.17 (0.98, 1.39) | 1.15 (0.98, 1.35) |
| **All-cause non-elective hospitalization** | 1286 (48.0) | 4348 (41.8) | 0.78 (0.71, 0.85) | 0.89 (0.81, 0.98) | 0.94 (0.89, 0.99) |
| **Stroke/TIA/systemic embolism** | 137 (5.1) | 467 (4.5) | 0.87 (0.72, 1.06) | 0.86 (0.70, 1.05) | 0.87 (0.72, 1.05) |
| **Any Bleeding** | 223 (8.3) | 762 (7.3) | 0.87 (0.75, 1.02) | 0.96 (0.81, 1.13) | 0.96 (0.83, 1.11) |
| **Composite of all-cause mortality or thromboembolic events** | 296 (11.0) | 1122 (10.8) | 0.97 (0.85, 1.11) | 1.05 (0.91, 1.21) | 1.04 (0.92, 1.18) |
| **Emergency department visits** | 2100 (78.4) | 6720 (64.6) | 0.50 (0.46, 0.56) | 0.59 (0.53, 0.66) | 0.87 (0.84, 0.90) |
| **Office-based physician visits** | 2657 (99.2) | 10342 (99.4) | 1.45 (0.89, 2.37) | 0.86 (0.50, 1.48) | 0.999 (0.99, 1.00) |
| **Older patients (age 65+) without any warfarin exposure in 90 days prior or 365 days post AF diagnosis** | | | | | |
| **All-cause mortality** | 179 (18.0) | 702 (18.2) | 1.01 (0.85, 1.22) | 0.99 (0.82, 1.21) | 0.995 (0.84, 1.17) |
| **All-cause non-elective hospitalization** | 622 (62.5) | 1974 (51.1) | 0.63 (0.54, 0.72) | 0.65 (0.55, 0.77) | 0.83 (0.76, 0.90) |
| **Stroke/TIA/systemic embolism** | 63 (6.3) | 206 (5.3) | 0.83 (0.62, 1.12) | 0.85 (0.62, 1.16) | 0.86 (0.64, 1.15) |
| **Any Bleeding** | 70 (7.0) | 233 (6.0) | 0.85 (0.64, 1.12) | 0.88 (0.66, 1.17) | 0.89 (0.68, 1.16) |
| **Composite of all-cause mortality or thromboembolic events** | 219 (22.0) | 849 (22.0) | 0.999 (0.84, 1.18) | 0.97 (0.81, 1.16) | 0.98 (0.84, 1.12) |
| **Emergency department visits** | 795 (79.8) | 2701 (69.9) | 0.59 (0.50, 0.70) | 0.63 (0.52, 0.76) | 0.89 (0.84, 0.94) |
| **Office-based physician visits** | 952 (95.6) | 3694 (95.6) | 1.01 (0.72, 1.42) | 0.87 (0.59, 1.28) | 0.99 (0.97, 1.01) |

^a^ OR indicates odds ratio; CI, confidence interval; RR, relative risk; adjusted OR and RR control for age, gender, year of diagnosis, location of diagnosis, patient demographics, health system utilization and comorbidities.

Medications in 90 days prior to index were also considered for analysis involving only patients age 65 and older. Corrected RR's are provided to aid in interpretation as some of the event rates are relatively common and therefore the odds ratio does not approximate the rate ratio. Calculations were based on the formula RR=OR/((1 - Po) + (Po x OR)), where Po is the event rate in the reference group (rural).
